# Antibiotic prescribing patterns for bacterial superinfection of mpox: A retrospective cohort study in an urban center

**DOI:** 10.1017/ash.2023.158

**Published:** 2023-06-29

**Authors:** William F. Simmons, Jeannie D. Chan, Jehan Z. Budak, Shireesha Dhanireddy, Margaret L. Green, Rupali Jain, Santiago Neme, Krista Rietberg, Alison C. Roxby, John B. Lynch, Chloe Bryson-Cahn

**Affiliations:** 1 Division of Allergy & Infectious Diseases, Department of Medicine, School of Medicine, University of Washington, Seattle, Washington; 2 Department of Pharmacy and School of Pharmacy, University of Washington Seattle, Washington; 3 Infection Prevention and Control, Harborview Medical Center, Seattle, Washington

## Abstract

Bacterial superinfection and antibiotic prescribing in the setting of the current mpox outbreak are not well described in the literature. This retrospective observational study revealed low prevalence (11%) of outpatient antibiotic prescribing for bacterial superinfection of mpox lesions; at least 3 prescriptions (23%) were unnecessary.

Human mpox, a zoonosis caused by the monkeypox virus, previously caused sporadic outbreaks originating in Africa with occasional travel-related spread. In 2022, a global outbreak drove significant spread outside endemic areas, with transmission among sexual partners.

The current outbreak has primarily affected men who have sex with men and people with human immunodeficiency virus (HIV).^1–5^ Unlike the classic cutaneous rash, many patients presented with oral or anogenital lesions. Mpox lesions are frequently vesiculopustular in nature, and bacterial skin and soft-tissue infection (SSTI) is often in the differential diagnosis.^3,5^

Several cases of bacterial superinfection of mpox lesions have been reported in this outbreak, but most case series only captured hospitalized patients with SSTI prevalence ranging from 3% to 11%.^1–6^ These rates are lower than those reported in previous outbreaks in endemic regions, where bacterial superinfection ranged from 19% to 47%.^7,8^ We conducted a retrospective chart review to assess secondary bacterial infection and antibiotic prescribing among patients with mpox.

## Methods

In this retrospective observational study, we reviewed the charts of all patients with mpox (defined as positive polymerase chain reaction [PCR] or tecovirimat prescription) seen at University of Washington Medicine between July 1, 2022, and August 31, 2022. UW Medicine includes a quaternary medical center, an urban safety-net hospital, a secondary inpatient facility, 3 emergency departments, and a network of clinics. Study data were collected using REDCap electronic data capture.^9^ The University of Washington Institutional Review Board approved the study and waived informed consent (no. STUDY00016342).

Clinical data included age, race, ethnicity, sex, microbiologic data, antibiotic prescriptions, and clinical outcomes. Data from other healthcare systems were obtained through linked electronic medical records. Risk factors for severe disease or lesions in anatomical areas constituting a special hazard were categorized based on US Centers for Disease Control and Prevention (CDC) guidance.

Antibiotic prescribing data was collected in the 2 weeks preceding and 4 weeks following mpox diagnosis (or tecovirimat prescription if results unavailable). Bacterial superinfection was defined as SSTI that was possibly associated with mpox lesions and treated with antibiotics. Sexually transmitted infection (STI) test results, location of possible bacterial infection, and infection collocation with mpox lesions were extracted.

The appropriateness of antibiotics for SSTI was determined by independent chart review by 2 investigators (W.S. and C.B.C.); a third investigator (J.B.L.) adjudicated discordant determinations. If the provider indicated clinical suspicion for bacterial superinfection or clinical reasoning was unavailable, the prescription was categorized as appropriate. If the provider indicated clinical findings as most consistent with mpox, or antibiotics were stopped once mpox was diagnosed, antibiotics were deemed inappropriate.

## Results

We identified 184 patients with mpox. However, 63 of these patients were excluded: 21 patients were identified from our center being the regional source for tecovirimat but were not cared for in our health system; 42 patients had insufficient data. Demographic and clinical data for included patients are listed in Table 1. Among the 6 patients who required hospitalization; 3 patients had dysphagia or pharyngitis, 1 patient had knee effusion, 1 patient had significant perineal edema, and 1 patient had facial edema.

**Table 1.** Demographic and Clinical Characteristics of Participants

Characteristic	Participants (n = 121), No. (%)^a^
Age, y (range)	37 (21–62)
**Sex**	
Male	120 (99)
Female	1 (1)
**Race**	
White	77 (64)
Black	13 (11)
>1 race	5 (4)
American Indian/Alaska Native	4 (3)
Asian	3 (2)
Native Hawaiian or Other Pacific Islander	2 (2)
Unavailable or declined	17 (14)
**Ethnicity**	
Hispanic or Latino	37 (31)
Not Hispanic or Latino	74 (61)
Unavailable or declined	10 (8)
**Risk factors for severe disease**	
Well controlled HIV (CD4>200 and VL<200)	71 (59)
Poorly controlled HIV (CD4<200 or VL>200)	10 (8)
Immunocompromise (non-HIV)	3 (2)
Atopic dermatitis	1 (1)
Other exfoliative skin conditions	5 (4)
**Lesions in areas that might constitute a special hazard**	
Anal/rectal/genital alone	74 (61)
Oral alone	10 (8)
Ocular alone	1 (1)
Oral and anal/rectal/genital	11 (9)
Ocular and anal/rectal/genital	1 (1)
Prescribed tecovirimat	112 (93)
Required hospitalization	6 (5)

Note. HIV, human immunodeficiency virus; CD4, T-lymphocyte; VL, viral load.

a Units unless otherwise indicated.

Moreover, 13 patients (11%) received antibiotics for presumed bacterial SSTI potentially associated with mpox lesions (Table 2). SSTI antibiotics were deemed inappropriate in 3 patients (23%). Also, 10 patients (77%) were started on SSTI antibiotics at the time of or prior to mpox testing, whereas 2 patients (15%) were prescribed systemic antibiotics and 1 patient (7%) was prescribed topical antibiotics after mpox diagnosis.

**Table 2.** Clinical Information of Patients Treated With Antibiotics for Suspected Bacterial Skin/Soft-Tissue Infection of Mpox Lesions

Age, y	Risk Factors for Severe Disease	High Risk Lesions	Admitted	Mpox diagnosis to Antibiotic Treatment, Days	Antibiotic Prescribed	Location of SSTI	Clinical Features of Interest	Antibiotic Appropriateness
40	None	AG		0	Amoxicillin-clavulanate	Genital	Erythema of penile lesion	Appropriate
23	HIV, controlled; other exfoliative, other immunocompromise	AG	Y: perineal edema	0	Cephalexin	Genital	Soft tissue swelling in groin	Appropriate
40	HIV, controlled	AG		0	Ciprofloxacin + metronidazole	Perirectal	Perirectal collection on CT, treated empirically	Appropriate
43	HIV, controlled	AG		−3	Doxycycline	Rectal + Extremity	Empiric treatment prior to mpox diagnosis, limited documentation	Appropriate
56	None	O		0	TMP/SMX + cephalexin	Oral	Empiric treatment, mpox felt most likely	Inappropriate
22	None	O		0	TMP/SMX + cephalexin	Face	Empiric treatment, treating associated thrombophlebitis and lesions	Appropriate
27	None	O		0	Clindamycin->TMP/SMX + cephalexin	Oral	Empiric treatment, ABX stopped once mpox diagnosed	Inappropriate
34	HIV uncontrolled	O, AG	Y: facial edema	2	TMP/SMX + cephalexin	Face	Honey-crusting of lip lesion	Appropriate
32	HIV uncontrolled	O, AG	Y: pharyngitis	0	Amoxicillin-clavulanate	Face	Facial swelling	Appropriate
36	HIV, controlled			6	Mupirocin	Face	Facial pustule	Appropriate
39	HIV, controlled			8	TMP/SMX + cephalexin	Groin	Groin erythema at mpox lesion	Appropriate
48	HIV, controlled		Y: septic knee	0	TMP/SMX + cephalexin	Extremity	Knee effusion: mpox positive, culture negative	Appropriate
38	HIV, controlled			0	Levofloxacin + doxycycline	Face	Empiric treatment, ABX stopped once mpox diagnosed	Inappropriate

Note. ABX, antibiotics; SSTI, bacterial skin and soft-tissue infection; TM-SMX, trimethoprim/sulfamethoxazole; O, oral lesions; AG, anogenital lesions; HIV, human immunodeficiency virus; CT, computed tomography.

Five other patients were initially treated with antibiotics for bacterial pharyngitis: 3 with a positive rapid strep, 1 patient despite a negative rapid strep, and 1 patient empirically. One was tested for mpox at initial presentation, but the mpox diagnoses of the other 4 patients were delayed until new lesions led to re-evaluation.

In total, 26 patients (21%) were prescribed antibiotics for STIs. Overall, 22 patients received ceftriaxone, and 22 patients received doxycycline; 18 of these patients received both antibiotics. In 81% of patients treated for STI testing, either results returned negative after antibiotics were prescribed or testing was never performed.

Other antibiotic prescriptions in 1 patient each were for abscess associated with a site of drug injection, urinary tract infection, antibiotic eye drops after injury, and prophylaxis.

## Discussion

Antibiotic prescribing for bacterial SSTI was uncommon during mpox infection (11%), and at least 23% of prescribed antibiotics were unnecessary using a conservative definition of inappropriateness. Other series have reported similarly low prevalence of bacterial superinfection, though predominantly in hospitalized patients.^2–6^ Our data represent the largest comprehensive series of antibiotic prescribing for SSTI in mpox.

Approximately 77% of antibiotic prescriptions for SSTI were initiated for empiric treatment of lesions, before mpox testing results were available. All unnecessary prescriptions were empiric. This phenomenon has not been described previously and may be due to clinical overlap between SSTI and mpox. Conversely, only 2 patients in our cohort were prescribed systemic antibiotics after mpox test results were available, suggesting knowledge of mpox diagnosis helped limit unnecessary antibiotic prescribing. Despite this, only 2 patients had antibiotics discontinued once testing results were available. The rarity of re-presentation with SSTI after mpox diagnosis suggests that bacterial superinfection is rare. Rapidly available testing may reduce unnecessary antibiotic prescribing for this viral infection.

Unusual clinical manifestations likely triggered antibiotic prescription. Several patients were started on antibiotics due to atypical mpox lesions, particularly ulcerated lesions. Also, 2 patients presented with fluid collections that were treated as bacterial (knee effusion and perirectal collection). When the knee was sampled, bacterial cultures were negative, whereas monkeypox PCR was positive.

Atypical symptoms delayed mpox diagnosis, resulting in unnecessary antibiotics. Of 5 patients initially treated for bacterial pharyngitis, only 1 patient was simultaneously tested for mpox. The 4 remaining patients were only diagnosed after development of new lesions. Providers should be aware that oral symptoms in patients with sexual exposure could represent mpox and that a low barrier to testing may reduce delayed diagnosis and unnecessary antibiotics.

The CDC recently warned that widespread use of tecovirimat could promote resistance and that clinicians should reserve treatment for patients with severe presentations.^10^ If tecovirimat for mild-to-moderate infections is no longer available, clinicians may prescribe antibiotics as an alternative. Our finding of low prevalence of bacterial infection supports stewardship efforts to withhold unnecessary antibiotics.

Our study also captured antibiotic prescribing around STI. Most STI treatment courses in our study (81%) were empiric. Mpox/STI coinfection rates varied between studies (17%–76%), and knowledge of local trends inform the need for empiric STI courses.^1–3,5^

Our study had several limitations. Antibiotic prescriptions as a proxy for bacterial superinfection may not accurately estimate true prevalence. Also, 63 patients were excluded due to insufficient data, which, along with a low absolute number of prescriptions, may have introduced systematic bias. Some patients with bacterial superinfection may have presented to outside providers. We mitigated this through linked medical records, which include most systems in the region. Prescriptions without available clinical reasoning were deemed appropriate, potentially overestimating appropriateness. A high proportion of patients were treated with tecovirimat compared to prior case series.^3^ Tecovirimat could have accelerated resolution of lesions, limiting bacterial infection, or the availability of tecovirimat may have reduced a provider’s impulse to prescribe antibiotics.

In this study, antibiotic treatment for bacterial SSTI in patients with mpox was uncommon. True bacterial superinfection was rare, and antibiotic treatment for bacterial superinfection was inappropriate in some cases. Antibiotics were frequently prescribed empirically due to diagnostic uncertainty, highlighting the importance of available rapid diagnostic testing. Mpox represents another opportunity for antimicrobial stewardship in the setting of viral infection.
